# Supplementary material for: The microbiome biomarkers of pregnant women’s vaginal area predict preterm prelabor rupture in Western China
Source: Front Cell Infect Microbiol. 2024 Oct 31;14:1471027. doi: 10.3389/fcimb.2024.1471027 (PMC11560878; doi:10.3389/fcimb.2024.1471027)
Supplement: Supplementary file 1 [file DataSheet1.zip › compare_1/Community/Pieplot/phylum/sample/P26.phylum.pieplot.pdf]

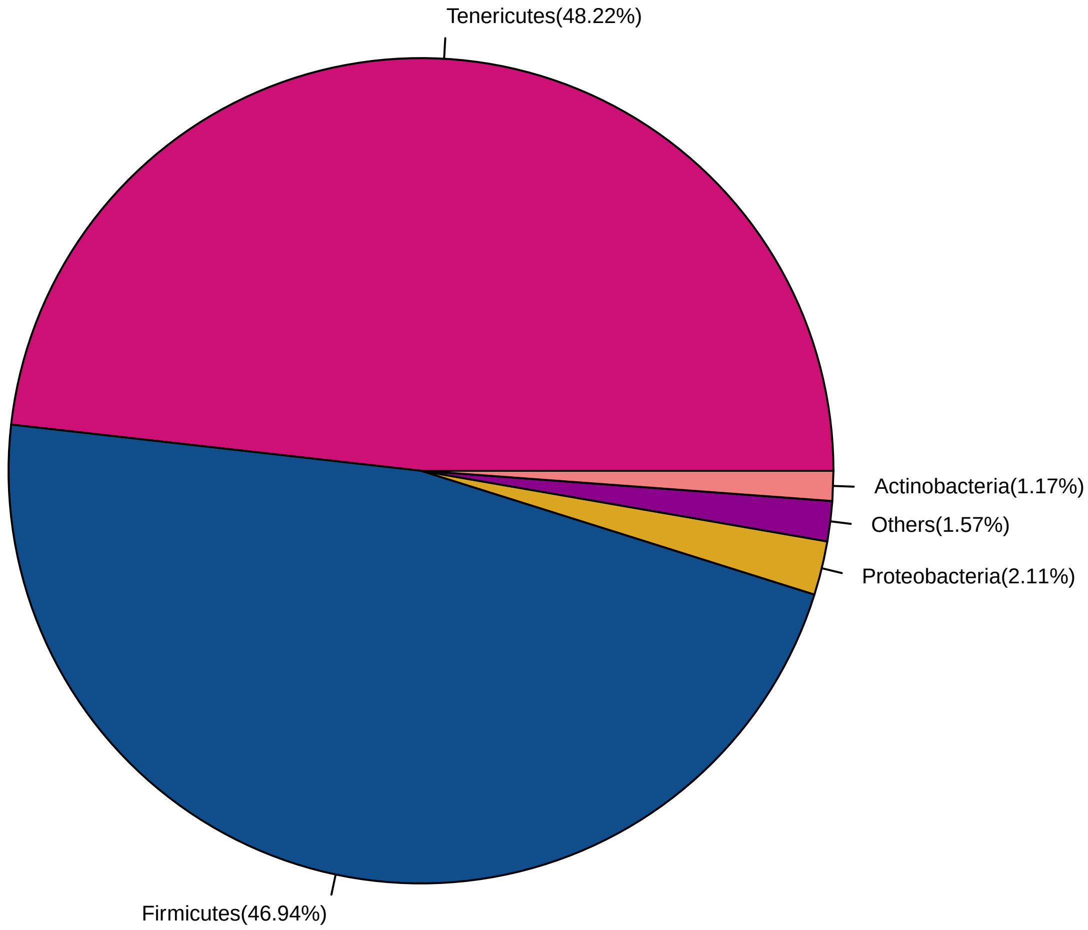
Tenericutes(48.22%)
Actinobacteria(1.17%)
Others(1.57%)
Proteobacteria(2.11%)
Firmicutes(46.94%)
